# Is the serotonin hypothesis/theory of depression still relevant? Methodological reflections motivated by a recently published umbrella review

**DOI:** 10.1007/s00406-022-01549-8

**Published:** 2023-02-15

**Authors:** Hans-Jürgen Möller, Peter Falkai

**Affiliations:** Department of Psychiatry and Psychotherapy, University Hospital, LMU Munich, Nussbaumstr. 7, 80336 Munich, Germany

The serotonin hypothesis of depression was first proposed in 1967, when the first antidepressants were being developed. It was subsequently refined, but for a long time it was criticized as being too one-sided. Later, the hypothesis was replaced by complex neurobiological theories, e.g., the chemical imbalance theory, which included additional neurotransmitters [1]. Consequently, the critical findings of the recently published umbrella review by Joanna Moncrieff and colleagues [2], which claim to falsify the serotonin hypothesis, come as no surprise. The publication of these findings is a good reason to carefully examine the content and methodologies of research on this topic and the basic problems associated with falsifying hypotheses and theories. However, for reasons of space, this editorial will discuss only a few of the main methodological aspects.

The umbrella review by Moncrieff et al. summarizes the results of all systematic reviews and meta-analyses on the serotonin hypothesis of depression and subdivides the hypothesis into six areas: serotonin and the 5-hydroxyindoleacetic acid (HIAA) level in body fluids, serotonin receptor activity, serotonin transporter activity, results of tryptophan depletion studies, serotonin transporter gene levels, and the interaction between the serotonin transporter gene and stress. The areas address the main serotonin theory but not all aspects of it. They make the complexity of the topic clear, in particular the fact that the serotonin theory comprises a bundle of related individual hypotheses. Thus, from the perspective of scientific theory, the authors have to confirm or falsify not a single hypothesis but a whole group of hypotheses held together by a complex theory. Testing and perhaps refuting such a complex theory is much more demanding than testing/refuting a single hypothesis.

The umbrella review includes only data from patients and no findings from animal experiments. The exclusion of animal studies limits the scope of the study considerably and is difficult to reconcile with the demands of testing a complex neurobiological theory.

In evidence-based medicine, meta-analyses and systematic reviews are considered to represent the highest evidence level. However, the inherent problems of these methodological approaches are often not adequately considered [3] and are also not discussed by Moncrieff et al. The main methodological problem of meta-analyses and thus also of the umbrella review is the question of how to decide which studies to include and which to exclude. Systems of formalistic rules exist for selecting studies, but content-related criticisms about study selection are frequently expressed by people with knowledge of the topic (e.g. clinical psychopharmacologists and neuroscientists). One wonders why from among the 360 studies identified by the PRISMA search process as being theoretically relevant, the systematic umbrella review included only 17 in its final evaluation/description. The respective flow diagram gives only a rough idea of the reasons why studies were excluded.

As is the case in many systematic reviews and meta-analyses, the content-related problems of the individual included studies are not discussed. However, these problems should be reviewed critically. It only makes sense to include studies that were well planned and implemented not only with respect to the formal aspects considered by systematic reviews and meta-analyses, but also with respect to their content. Whether and how this latter aspect was assessed remains unclear in the umbrella review because the authors do not discuss it in detail. Therefore, one must assume that such a detailed evaluation was performed not by the authors of the umbrella review but by the authors of the original meta-analyses. However, that was probably not the case in most of the meta-analyses because when selecting empirical studies for inclusion in such analyses, researchers normally check only formal aspects. The aspects that should actually be considered when evaluating studies were presented by Riederer [4], among others, by using the example of studies related to serotonin/tryptophan and include the following: problems in determining serotonin in plasma HIAA in cerebrospinal fluid; the fact that plasma serotonin does not reflect the serotonin concentration in the brain because serotonin is metabolized at the blood–brain barrier; consideration of the suboccipital/lumbar HIAA gradient when performing a lumbar puncture; and the temporal difference between tryptophan depletion and the effects of serotonin metabolism on the brain.

In addition to systematic reviews and meta-analyses on the serotonin hypothesis, the umbrella review includes several large studies and a large genetic study based on UK-wide data; the former studies summarize data from individual studies without using the strict approach of a systematic review. The authors state that including these studies was the best way to comprehensively portray the evidence. However, this approach is unusual for an umbrella review and methodologically questionable. Although umbrella reviews typically consider previous meta-analyses/systematic review of primary studies and umbrella reviews of meta-analyses/systematic reviews (also termed “meta-umbrella reviews”) separately, Moncrieff et al. summarized these different types of studies together. This approach means that a comparison of effect sizes is potentially unreliable. In addition, the selection criteria for the primary studies are unclear, which opens the door to uncontrolled selection biases: Some primary studies appear to have been included at the expense of others.

The tryptophan-related studies can be used as an example of how problematic the presentation by Moncrieff et al. is in terms of study selection [5]. Moncrieff et al. included one meta-analysis, one systematic review, and ten recent studies involving healthy volunteers, but they did not include a clinical and molecular imaging study that showed an effect in people with major depressive disorder [6]. They also omitted several studies included in two meta-analyses that evaluated circulating concentrations of tryptophan, a substance that directly influences central serotonin [7, 8].

The umbrella review also contains a number of material errors and misinterpretations, e.g., concerning imaging data on both 5-HT_1A_ receptor and serotonin transporter protein (SERT) binding [5]. For example, the statement by Moncrieff et al. [2] that 5HT_1A_ receptors are known as autoreceptors mistakenly assumes that 5HT_1A_ receptors are exclusively pre-synaptic autoreceptors, whereas most of these receptors are post-synaptic 5-HT_1A_ heteroreceptors. Reduced availability of post-synaptic 5-HT_1A_ receptors in unmedicated depression would be consistent with decreased 5-HT neurotransmission.

Overall, the included studies in the various relevant areas produced hardly any evidence for the serotonin theory, and at the most, they found weak connections that support only a few aspects of it. Therefore, the authors conclude from their results that their evaluation cannot confirm the serotonin theory of depression. Even though the authors discuss some of the methodological problems of the individual studies and meta-analyses and the reason for the negative results, they believe that their overall result falsifies the serotonin hypothesis, in particular because they consider the umbrella review approach, which summarizes all available reviews and meta-analyses, as the highest level of evidence synthesis.

Even if one initially accepts the result of the umbrella review by Moncrieff et al. [2], the broad non-confirmation of various sub-hypotheses of the serotonin theory of depression does not mean that the theory is completely false and, consequently, that a neurobiological explanation of depression is refuted. As in other areas of medicine, the serotonin theory has been expanded through various new basic research findings, e.g., neurogenesis and synaptogenesis, neuronal networks, neuroendocrinology, neuroinflammation, and genetics, independent of the serotonergic system, so these aspects must be included in the etiopathological reflections on the cause of the complex disease depression or its subgroups [1]. Consequently, the studies on the serotonergic system included in the umbrella review represent only part of the complex neurobiological understanding of depression. The serotonin theory can definitely continue to be scientifically relevant as a partial aspect of the theoretical concept of depression and as part of more complex concepts.

Of relevance in this context is the work of the famous science theorist Thomas S. Kuhn, who showed in his studies on the history of science [9] that in contrast to the falsification theory from the equally famous science theorist Karl Popper—whom we are in no way questioning here—most complex theories, unlike simple hypotheses, cannot be refuted by falsification. Instead, people lose interest in them because of paradigm shifts in the sense that a younger generation of researchers becomes interested in other theories or because highly complex theories characterized by more advanced technologies gain the upper hand. However, until that happens, the theories can retain a certain usefulness for research, even if they are insufficiently proven. These considerations help one understand why, despite being criticized, the serotonin theory is still relevant as an explanation of depression. Depression is characterized neurobiologically by an imbalance of a complex dynamic system involving genetic, epigenetic, environmental, and stress vulnerabilities, and this imbalance initiates a cascade of neurobiological alterations in and beyond serotonergic functioning. All integrally related pathways interact multi-directionally throughout the various phases of depression [1].

Moncrieff et al. do not limit their argumentation to their critical conclusion about the validity of the serotonin theory of depression. Instead, with the following sentence in the Discussion section they go far beyond the empirical results of their study by drawing conclusions about the use of antidepressant treatment: “The idea that depression is the result of a chemical imbalance also influences decisions about whether to take or continue antidepressant medication and may discourage people from discontinuing treatment, potentially leading to lifelong dependence on these drugs” (2, p. 11). This treatment-related conclusion is highly problematic and cannot be directly inferred from the result of the umbrella review. Furthermore, the efficacy of antidepressant treatment, including serotonergic antidepressants, is well supported by the evidence [10]. In principle, we can view the efficacy of antidepressants independently from the validity of the serotonin theory of depression and simply interpret the serotonin mechanism as a favorable mechanism for achieving antidepressant effects, not as a theory of causality of depression. Interestingly, Moncrieff et al. do not cite studies that prove the efficacy of antidepressants; as far as the serotonergic antidepressants are concerned, such studies could well be discussed as providing ex juvantibus support for the serotonin theory.

The conclusion drawn by Moncrieff et al. [2] that the usefulness of antidepressant treatment should be questioned—a conclusion that is in line with the position that Joanna Moncrieff has frequently published, especially in the lay press—can have severe negative consequences for the treatment adherence of people with depression. The question we need to ask is whether the results of their umbrella review are strong enough, as far as the methodology and content of the review are concerned, that they allow such a far-reaching conclusion to be drawn or whether this conclusion rather reflects the authors’ own bias.

## Data Availability

Not applicable.
